# Synergistic Effect between Ultra-Small Nickel Hydroxide Nanoparticles and Reduced Graphene Oxide sheets for the Application in High-Performance Asymmetric Supercapacitor

**DOI:** 10.1038/srep11095

**Published:** 2015-06-08

**Authors:** Yonghuan Liu, Rutao Wang, Xingbin Yan

**Affiliations:** 1 Laboratory of Clean Energy Chemistry and Materials, State Key Laboratory of Solid Lubrication, Lanzhou Institute of Chemical Physics, Chinese Academy of Sciences, Lanzhou 730000, P. R. China; 2 Graduate University of Chinese Academy of Sciences, Beijing 100080, P. R. China

## Abstract

Nanoscale electrode materials including metal oxide nanoparticles and two-dimensional graphene have been employed for designing supercapacitors. However, inevitable agglomeration of nanoparticles and layers stacking of graphene largely hamper their practical applications. Here we demonstrate an efficient co-ordination and synergistic effect between ultra-small Ni(OH)_2_ nanoparticles and reduced graphene oxide (RGO) sheets for synthesizing ideal electrode materials. On one hand, to make the ultra-small Ni(OH)_2_ nanoparticles work at full capacity as an ideal pseudocapacitive material, RGO sheets are employed as an suitable substrate to anchor these nanoparticles against agglomeration. As a consequence, an ultrahigh specific capacitance of 1717 F g^−1^ at 0.5 A g^−1^ is achieved. On the other hand, to further facilitate ion transfer within RGO sheets as an ideal electrical double layer capacitor material, the ultra-small Ni(OH)_2_ nanoparticles are introduced among RGO sheets as the recyclable sacrificial spacer to prevent the stacking. The resulting RGO sheets exhibit superior rate capability with a high capacitance of 182 F g^−1^ at 100 A g^−1^. On this basis, an asymmetric supercapacitor is assembled using the two materials, delivering a superior energy density of 75 Wh kg^−1^ and an ultrahigh power density of 40 000 W kg^−1^.

Recently, supercapacitors have become promising candidates in energy storage systems owing to their extreme high power performance, moderate energy density and excellent cycle life^1^,^2^,^3^,^4^,^5^. Based on the charge storage mechanism, supercapacitors are mainly divided into electrical double layer capacitors (EDLCs), which store energy based on the reversible adsorption/desorption of ion at the interface between the electrode materials and the electrolyte, and pseudocapacitors capacitors, which store energy from rapid surface redox reactions on the surface and the near surface of the electrode materials^6^,^7^,^8^. In this regard, the electrochemical performance of supercapacitors directly relates to electrode materials, such as carbon materials, transition-metal oxides and conducting polymers^9^,^10^. To the best of our knowledge, the characteristic dimensions of the electrode materials are quite important for supercapacitors performance, especially for rate capability, since the diffusion time of electrolyte ions (*t*) is proportional to the square of the diffusion length (*L*) (*t* *≈* *L*^*2*^*/D*)^11^,^12^,^13^. Based on above analysis, a great of endeavors have been focused on preparing small size^14^, porous^15^, thin^16^,^17^, and hollow spheres^18^ with short of the diffusion distance and exposure of abundant active sites for improving electrochemical performance. For pseudocapacitors, Ni(OH)_2_ has been recognized as one of the most attractive pseudocapacitive materials because of its easy synthesis, low cost and high theoretic capacitance^19^,^20^,^21^. Hence, the design and synthesis of the nano-sized Ni(OH)_2_ for high-performance supercapacitors are attractive. However, the nanoscale-particles, especially when the particle size is under 10 nm, can self-assemble into large pieces or bulk owing to their high interface energy^22^. Therefore, the fully utilization of the ultra-small pseudocapacitive materials for supercapacitors is challenging.

In general, a simple strategy to prevent the agglomeration and improve the conductivity of the nano-sized pseudocapacitive materials is to combine them with the highly conductive support materials^23^,^24^. For example, graphene, a typical two-dimension planar structure material, can be considered as the most attractive substrate for pseudocapacitive materials owing to its large surface area, high conductivity and excellent mechanical property^19^,^20^,^21^. In view of its virtue, various nanomaterials-graphene composites have been widely reported for electrochemical energy storage^24^,^25^,^26^. The results indicate that the scientific design of hybrid structures can obviously improve electrochemical performance because graphene sheets serve as a substrate not only to prevent nanomaterials from aggregating by balancing their high interface energy^27^,^28^, but also to offer an efficient electron transport pathway^25^,^26^.

It is well known that graphene sheets are an ideal electrical double layer capacitor material. However, they suffer from irreversible sheet-to-sheet restacking due to the strong interlayer van der Waals force under drying and heat treatment processes, resulting in a limit of the accessibility to electrolyte ions and largely hindering the full utilization of their surfaces and active sites^29^,^30^. Recently, a facile strategy to reduce agglomeration of reduced graphene oxide (RGO) sheets for improving their electrochemical performance is to introduce interlayer ‘spacers’, including nanoparticles^10^, nanotubes/nanowires^31^,^32^ and nanosheets^33^ among the graphene layers.

Herein, by taking advantage of each merits, the synergistic effect between ultra-small Ni(OH)_2_ nanoparticles and RGO sheets for designing high-performance asymmetric supercapacitor (ASC) is demonstrated. RGO sheets are employed as an suitable substrate to anchor the Ni(OH)_2_ nanoparticles against their agglomeration, and the ultra-small Ni(OH)_2_ particles act as recyclable sacrificial spacers within RGO layers to effectively reduce the irreversible restacking of RGO sheets, leading to higher specific surface area and superior rate performance due to faster ion diffusion compared to pure Ni(OH)_2_ nanoparticles and initial RGO, respectively. Consequently, the as-prepared RGO-Ni(OH)_2_ composite exhibits excellent specific capacitance of 1717 F g^−1^ at 0.5 A g^−1^ and the enhanced RGO sheets exhibit superior rate capability with a high specific capacitance of 182 F g^−1^ at an ultrahigh rate of 100 A g^−1^. Finally, by using the two materials as the positive and negative electrodes, a high-performance ASC with a superior energy density (75 Wh kg^−1^) and an ultrahigh power density (40 000 W kg^−1^) is achieved.

## Results

### Positive electrode materials of Ni(OH)_2_ and RGO-Ni(OH)_2_ composite

**Figure 1:**
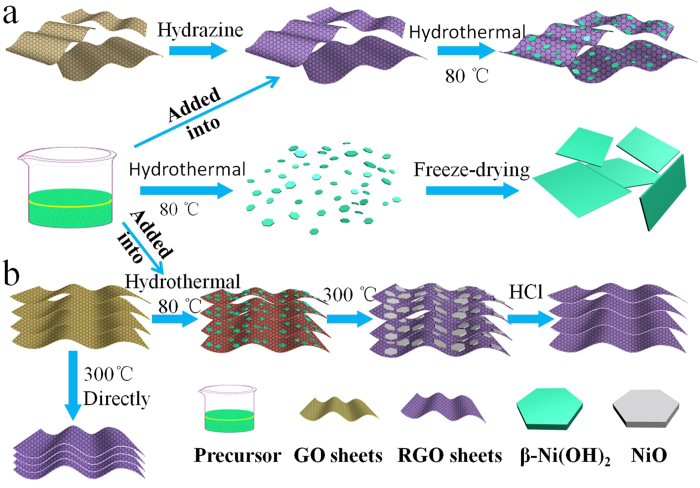
Schematic illustration of the synthesis process: (**a**) the RGO-Ni(OH)_2_ composite and pure Ni(OH)_2_, and (**b**) the enhanced RGO sheets with Ni(OH)_2_ particles as spacers and the conventional RGO sheets prepared by directly thermal treatment.

**Figure 2:**
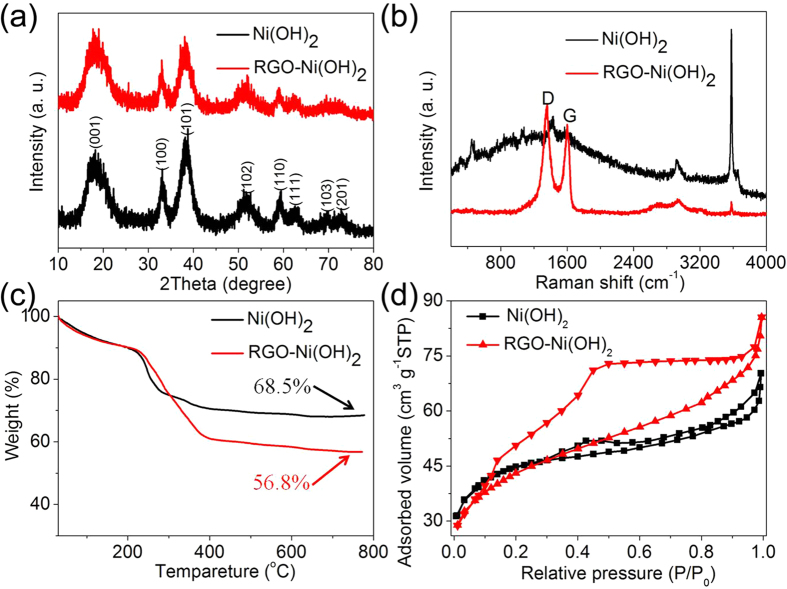
(**a**) XRD patterns, (**b**) Raman spectra, (**c**) TG curves and (**d**) Nitrogen adsorption/desorption isotherms of Ni(OH)_2_ and RGO-Ni(OH)_2_ composite.

The preparation process of Ni(OH)_2_ and RGO-Ni(OH)_2_ (denoted as RGO-Ni(OH)_2_-2 in Supplementary Information) composite is displayed in Fig. 1, and other samples with different mass ratio of RGO to RGO-Ni(OH)_2_ are also shown (see Supplementary table S1). Figure 2A shows the XRD patterns of as-prepared RGO-Ni(OH)_2_ composite, together with the pure Ni(OH)_2_ sample for comparison. The diffraction pattern of pure Ni(OH)_2_ sample exhibits a set of characteristic diffraction peaks, and all of them can be indexed to the standard data for hexagonal phase of β-Ni(OH)_2_ (JCPDS card no. 14-0117), which is well-agreement with the reported pattern for hexagonal β-Ni(OH)_2_^34^,^35^. In comparison, the XRD pattern of the RGO-Ni(OH)_2_ composite is similar to that of pure Ni(OH)_2_, indicating that Ni(OH)_2_ with high purity can be well prepared on the RGO. It should be noted that RGO-Ni(OH)_2_ composite shows a poor crystallinity of β-Ni(OH)_2_, which may be in favor of enhancing the electrochemical performance^36^. In addition, the typical features of graphene can be found in the following Raman spectra, SEM and TEM images.

The Raman spectra of pure Ni(OH)_2_ and the RGO-Ni(OH)_2_ composite are displayed in Fig. 2b. Two prominent peaks at 1349 cm^−1^ and 1603 cm^−1^ in the spectrum of the RGO-Ni(OH)_2_ composite can be attributed to the D and G bands of RGO, respectively. Generally, the G band represents the in-plane bond-stretching motion of the pairs of C sp^2^ atoms (the E_2g_ phonons). The strong D band implies a large number of defects of as-prepared RGO, which mainly results from the weak reduction way. Commonly, the peak intensity ratio of the D and G band can be used to roughly estimate the disorder degree and average size of the sp^2^ domains of the graphite materials^18^,^37^. Thus, the high ratio (I_D_/I_G_ = 1.38) indicates there are a lot of defects of the as-prepared RGO. Moreover, the peaks at 3591 cm^−1^ and 2923 cm^−1^ can be assigned to the characteristic peaks of β-Ni(OH)_2_^38^,^39^. The result further confirms that Ni(OH)_2_ has been successfully anchored on the RGO sheets.

The carbon content of the RGO-Ni(OH)_2_ composite can be determined by the TGA technique. Figure 2c displays the representative TGA curves of pure Ni(OH)_2_ and RGO-Ni(OH)_2_ composite. The two samples both show a slight weight change below 230 °C because of the evaporation of adsorbed water molecules^40^,^41^. The evident weight loss of pure Ni(OH)_2_ at the temperature range 240–300 °C comes from the decomposition of Ni(OH)_2_ to NiO. Nevertheless, the obvious weight change of RGO-Ni(OH)_2_ composite at the temperature change from 240 °C to 390 °C is attributed to both the decomposition of Ni(OH)_2_ to NiO and the combustion of the RGO. Based on the residual weight of Ni(OH)_2_ and RGO-Ni(OH)_2_ about 68.5 wt% and 56.8 wt%, respectively, which reveals that the mass percentage of RGO in the RGO-Ni(OH)_2_ composite is 14.9 wt%. This value is consistent with the result (14.0%) from elemental analysis (vario EL, see Supplementary table S1).

Figure 2d shows nitrogen adsorption/desorption isotherms of the pure Ni(OH)_2_ and RGO-Ni(OH)_2_ composite samples at 77 K. The corresponding isotherm curves in the range of 0.3–0.9 P/P_0_ are characteristics of mesoporous materials. The BET surface area of RGO-Ni(OH)_2_ composite is 152 m^2^ g^−1^ which is higher than that of the pure Ni(OH)_2_ sample (109 m^2^ g^−1^). The pore size distribution (see Supplementary Fig. S1) of pristine Ni(OH)_2_ shows very thin micropores and mesopores ranging from 1.5 to 2.4 nm, and a small number of macropores in the range of 147–185 nm. Compared to pure Ni(OH)_2_, RGO-Ni(OH)_2_ composite shows wider pore size distribution of 1.5–68 nm and 120–160 nm. These considerable mesopores and macropores can facilitate the rapid migration of electrolyte ions during the charge/discharge process, which is highly crucial for high-performance supercapacitors^20^,^37^.

**Figure 3:**
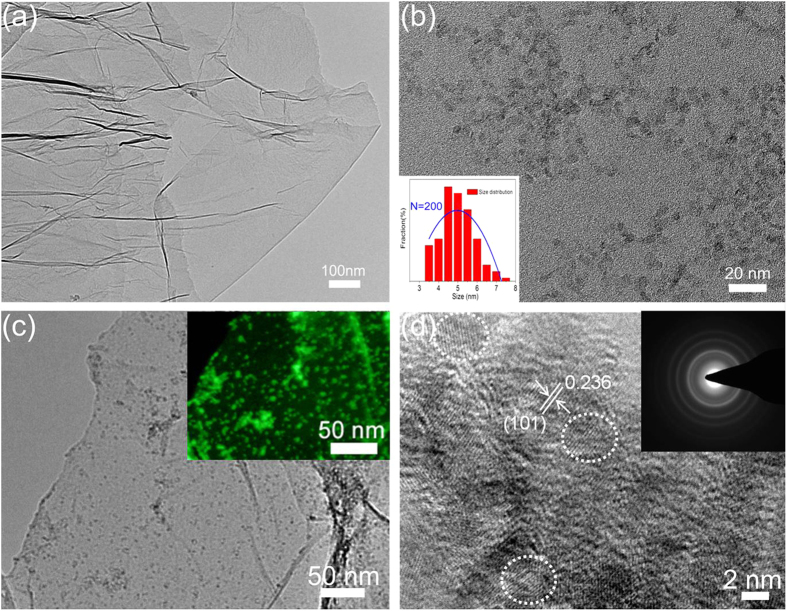
(**a**) TEM image of the RGO. (**b**) TEM image and the corresponding size distribution of the Ni(OH)_2_ particles. (**c**) TEM image of the RGO-Ni(OH)_2_ composite. Inset shows an annular dark field scanning TEM (ADF-STEM) image of RGO-Ni(OH)_2_ composite. (**d**) High-resolution TEM image of the RGO-Ni(OH)_2_ composite and SAED image of Ni(OH)_2._

**Figure 4:**
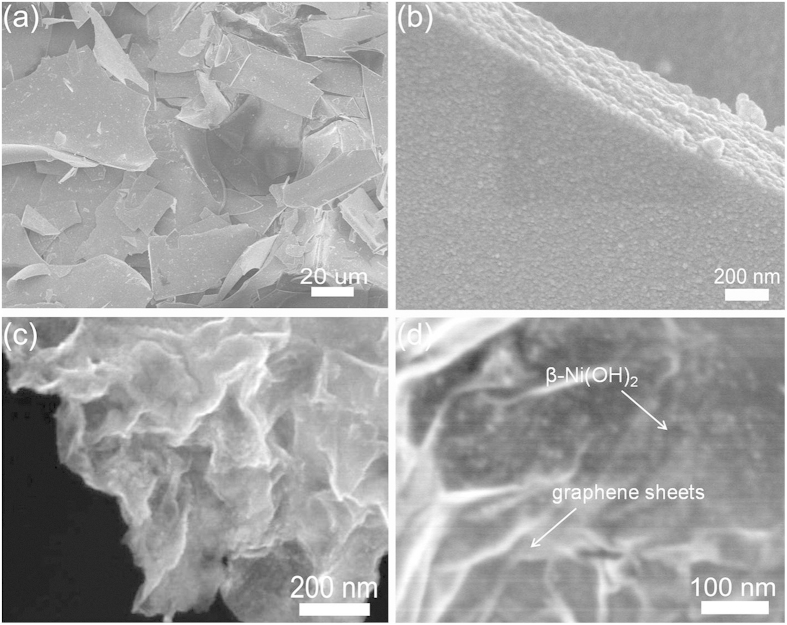
SEM images of the pure Ni(OH)_2_ and RGO-Ni(OH)_2_ composite: (**a** and **b**) low and high magnification SEM images of Ni(OH)_2_ sheets, and (**c** and **d**) low and high magnification SEM images of RGO-Ni(OH)_2_ composite.

The detailed morphology and structural properties of Ni(OH)_2_ and RGO-Ni(OH)_2_ composite are examined by TEM and SEM, as shown in Fig. 3 and Fig. 4. As displayed in Fig. 3a, the RGO sheets are quite thin and possess a small number of wrinkles, indicating that they can act as ideal substrates to support the ultra-small Ni(OH)_2_ particles. From Fig. 3b, it can be seen that the pristine Ni(OH)_2_ nanoparticles have a uniform size with the average size of 4.8 nm. However, from the SEM images shown in Fig. 4a,b, these pristine nanoparticles would be interconnected with each other to randomly form big disordered sheets with several tens micrometer length and hundreds of nanometer thickness after freeze-drying. In comparison, Fig. 3c,a annular dark field scanning TEM (ADF-STEM) image (inset) shows that, after incorporation of RGO sheets, the ultra-small Ni(OH)_2_ particles are high-dispersedly grown on RGO sheets with mean size of 4.6 nm. Naturally, a small number of particle aggregations mainly result from the wrinkles or overlaps of RGO sheets. Moreover, seen from the SEM images of RGO-Ni(OH)_2_ composite given in Fig. 4c,d, the ultra-small Ni(OH)_2_ particles with uniform size of 5.1 nm are relatively homogeneously anchored on RGO sheets. In the HRTEM image of composite shown in Fig. 3d, the measured lattice spacing of about 0.236 nm is well consistent with the value calculated from the XRD data of the (101) crystalline plane of the β-Ni(OH)_2_. Besides, the selected area electronic diffraction pattern (SAED) shows some well-defined rings that reveal the ultra-small Ni(OH)_2_ on RGO with a great polycrystalline nature (inset of Fig. 3d).

**Figure 5:**
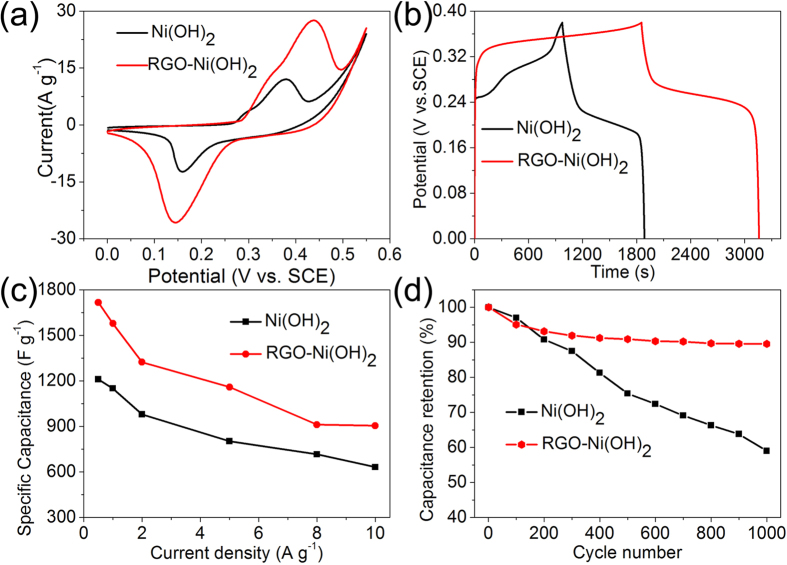
(**a**) CV curves of pure Ni(OH)_2_ and RGO-Ni(OH)_2_ composite at a scan rate of 5 mV s^−1^. (**b**) Galvanostatic charge and discharge curves of pure Ni(OH)_2_ and RGO-Ni(OH)_2_ composite at a current density of 0.5 A g^−1^. (**c**) The specific capacitance as a function of current density for pure Ni(OH)_2_ and RGO-Ni(OH)_2_ composite. (**d**) Cycling performance of pure Ni(OH)_2_ and RGO-Ni(OH)_2_ composite at 20 mV s^−1^ in 2.0 M KOH electrolyte.

The electrochemical performance of as-prepared pure Ni(OH)_2_ and RGO-Ni(OH)_2_ composite were evaluated by Cyclic Voltammetry (CV), galvanostatic charge/discharge (GCD) and electrochemical impedance spectroscopy (EIS) in a three-electrode cell with 2 M KOH aqueous electrolyte. Figure 5a shows the typical CV curves of as-prepared samples at a scan rate of 5 mV s^−1^, which consists of a pair of strong reversible redox peaks, indicating that the capacitance is a typical of pseudocapacitance behavior. The reaction process can be simply expressed as follows:


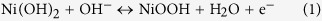


Generally, the specific capacitance of the electrode is directly proportional to its CV area. Based on the CV curves depicted in Fig. 5a, we can easily figure out the capacitance of RGO-Ni(OH)_2_ composite is much higher than that of the pure Ni(OH)_2_. In addition, the samples with other ratios of RGO to RGO-Ni(OH)_2_ were evaluated by CV measurements as well (see Supplementary Fig. S2), which all exhibit typical pseudocapacitance behaviors at low and high scan rates.

Galvanostatic charge/discharge (GCD) measurements were adopted to study the capacitance performance and rate capability of Ni(OH)_2_ and RGO-Ni(OH)_2_ composite. Their GCD curves performed in a potential window of 0–0.38 V at a current density of 0.5 A g^−1^ are depicted in Fig. 5b. Clearly, RGO-Ni(OH)_2_ composite exhibits a higher specific capacitance of 1717 F g^−1^ at 0.5 A g^−1^, higher than that of pure Ni(OH)_2_ (1210 F g^−1^). Furthermore, from Fig. 5c, RGO-Ni(OH)_2_ composite shows much better capacitances performance compared to pure Ni(OH)_2_ at various current densities. At different discharge current densities of 1, 2, 5, 8, 10 A g^−1^, the specific capacitance of RGO-Ni(OH)_2_ are 1578, 1323, 1159, 912 and 905 F g^−1^, respectively. For pure Ni(OH)_2_, the corresponding results are 1150, 979, 802, 715 and 631 F g^−1^, respectively. The more detailed discharge measurements of as-prepared electrode materials were fully researched (see Supplementary Fig. S3). It is clearly seen that the rate performance of Ni(OH)_2_ is largely improved after introduction of RGO, which is benefiting from two main reasons: on one hand, the ultra-small Ni(OH)_2_ particles with poor crystallinity high-dispersedly distribute on the surfaces of RGO, which can provide more active sites for faradaic redox and is in favor of ion contact and transfer^36^; on the other hand, the RGO can provide rapid electron transport paths for the fast faradaic reaction, which is the key for both the higher specific capacitance and better rate performance. While it is worth noting that there is a ratio balance between Ni(OH)_2_ and RGO for electrochemical performance, because of hardly capacitance contribution of RGO at this window of 0–0.55 V.

The Nyquist plots (see Supplementary Fig. S4) display electrochemical impedance spectroscopy (EIS) of pure Ni(OH)_2_ and RGO-Ni(OH)_2_ composite. It is clearly seen that the Nyquist plot of RGO-Ni(OH)_2_ composite shows smaller arc shape at the high frequency region and more vertical line at the low frequency region, indicating faster reaction kinetics.

Since the cycle stability is a quite crucial parameter for supercapacitors, the Ni(OH)_2_ and RGO-Ni(OH)_2_ composite were further tested by repeating CV tests at a scan rate of 20 mV s^−1^ for 1 000 cycles as shown in Fig. 5d. It is worth noting that the capacitance retention of RGO-Ni(OH)_2_ composite after 1 000 cycles is 89%, which is much higher than pure Ni(OH)_2_ only with 59%.

### Negative electrode materials of RGO and enhanced RGO

**Figure 6:**
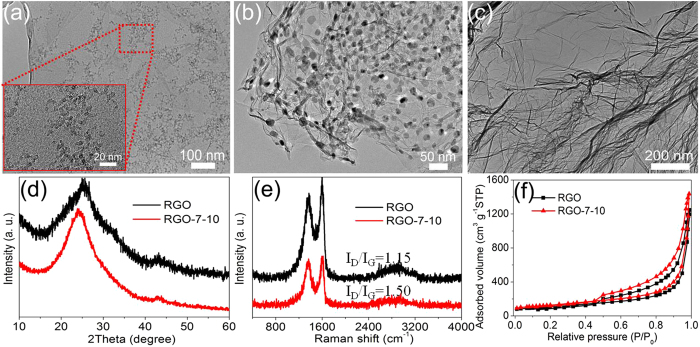
(**a**) TEM image of the GO-Ni(OH)_2_ composite. (**b**) TEM image of the RGO-NiO composite after thermal treatment. (c) TEM image of the enhanced RGO (RGO-7–10 sample) after HCl treatment. (**d**) XRD patterns, (**e**) Raman spectra and (**f**) Nitrogen adsorption/desorption isotherms of RGO and RGO-7–10.

After hydrothermal process of GO and Ni(OH)_2_ precursor mixture solution, it is clearly seen that the ultra-small Ni(OH)_2_ nanoparticles (Fig. 6a) grown on GO have similar size compared to above Ni(OH)_2_ nanoparticles anchored on the surfaces of RGO (Fig. 3c). After the subsequent thermal treatment, the Ni(OH)_2_ particles grown on RGO became NiO nanoparticles with a mean size of 14.6 nm (Fig. 6b). Importantly, after removing these NiO nanoparticles by HCl etching, as-obtained RGO-7–10 sample exhibits a distinctly crumpled morphology (Fig. 6c), and the high magnification TEM image (see Supplementary Fig. S5d) shows abundant ‘footprints’, revealing the important role of Ni(OH)_2_ and NiO nanoparticles as spacers to prevent the restacking of graphene sheets during the drying and the thermal treatment processes. For comparison, the high magnification TEM image of the pristine RGO is also given (see Supplementary Fig. S5c). Besides, as shown in SEM images of RGO and RGO-7–10 (see Supplementary Fig. S5a and b), the RGO exhibits a dense and frizzy layered structure because of its strong shrink and stacking. However, with the aid of Ni(OH)_2_/NiO nanoparticles as spacers to reduce stacking of RGO, the resulting RGO-7–10 shows distinctly laminar and crumpled morphology.

XPS and FITR were employed to examine chemical species of as-prepared RGO materials, and the results indicate the similar elements content and species in all samples (see Fig. S6 and Supplementary Table S2). It means that the adding of Ni(OH)_2_ nanoparticles in the preparation process does not obviously affect the chemical components of the final RGO products. XRD and Raman were used to further analyze the microstructure of the samples (Supplementary Fig. S6). Compared to RGO prepared by conventional direct thermal reduction without adding Ni(OH)_2_ nanoparticles, the peak (002) center of RGO-7–10 shifts down to about 24.1° (Fig. 6d), which reveals a highly disordered restacking of sheets, demonstrating a loose structure of RGO-7–10. In addition, Fig. 6e shows Raman spectra of RGO and RGO-7–10. I_D_/I_G_ ratio of RGO-7–10 is 1.50, which is much higher than RGO (1.15), indicating that there are more defects remaining on RGO-7–10, which can be attributed to the abundant wrinkles and the decrease in the number or average size of the sp^2^ graphene domains^18^,^37^. Fig. 6f shows N_2_ adsorption/desorption isotherms of RGO and RGO-7–10, which exhibits type isothermal curves with a distinct hysteresis loop at a relative pressure (p/p_0_) from 0.45 to 1.0, indicating the existence of a certain amount of mesopores. The RGO-7–10 has specific surface area of 443 m^2^ g^−1^, which is higher than RGO (363 m^2^ g^−1^). This is benefiting from that the introduction of Ni(OH)_2_ could reduce the agglomeration/restacking of RGO during the drying and thermal treatment processes.

**Figure 7:**
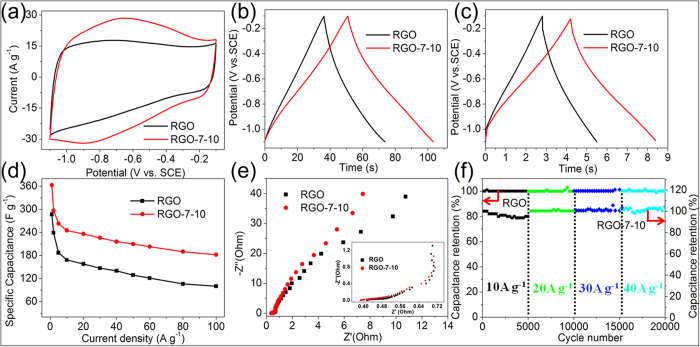
(**a**) CV curves of RGO and RGO-7–10 at a scan rate of 100 mV s^−1^. (**b** and **c**) GCD curves of RGO and RGO-7–10 at current densities of 5 and 50 A g^−1^, respectively. (**d**) The specific capacitance as a function of current density for RGO and RGO-7–10. (**e**) Nyquist plots of RGO and RGO-7–10. Inset shows the close-up view of the high-frequency regime. (**f**) Cycling stability of RGO and RGO-7–10 at different current densities of 10 A g^−1^, 20 A g^−1^, 30 A g^−1^ and 40 A g^−1^, respectively. The running cycle at each current density is the same as 5 000 cycles.

The electrochemical properties of as-prepared RGO samples were evaluated in a three-electrode cell with 2 M KOH aqueous electrolyte as well. The CV curves (Fig. 7a) show that CV area of RGO-7–10 is much more than RGO at a scan rate of 100 mV s^−1^. The difference area of CV curves mainly comes from redox reaction of residual oxygen-containing functionalities on the RGO^29^. For RGO-7–10, the pseudocapacitance is demonstrated more completely than RGO due to its wider distance among RGO sheets, which provides more active sites and facilitates the accessibility to electrolyte ions. From the GCD curves (Fig. 7b), RGO-7–10 exhibits a higher specific capacitance of 263 F g^−1^ at a current density of 5 A g^−1^ compared to RGO (187 F g^−1^). It is worth noting that the RGO-7–10 still has a large specific capacitance of 210 F g^−1^ at a high current density of 50 A g^−1^ (Fig. 7c), which is still much higher than RGO (129 F g^−1^). In addition, Fig. 7d shows rate performance of both electrode materials, we can clearly see that the specific capacitance and rate performance are both significantly improved for RGO-7–10. Even at a very high current density of 100 A g^−1^, RGO-7–10 still demonstrates a high specific capacitance of 182 F g^−1^. These capacitance values are higher than the similar materials in previous works, such as holey graphene nanosheets (HGNSs) (170 F g^−1^ at a current density of 50 A g^−1^)^42^, thermally reduced graphene oxide (200 F g^−1^ at a current density of 4 A g^−1^)^43^ and graphene-CNTs composite (269 F g^−1^ at a scan rate of 5 mV s^−1^)^44^. Moreover, for all RGO samples prepared with the aid of Ni(OH)_2_ nanoparticles, their capacitances at different rates are all higher than those of the RGO prepared by conventional direct thermal reduction (see Supplementary Fig. S7 and S8). We believe that the excellent capacitance and rate capability of RGO-7–10 are attributed to wider layer distance resulting from introduction of Ni(OH)_2_/NiO particles as spacers among RGO sheets (see the structural diagram in Fig. 1). Figure 7e shows EIS curves of RGO and RGO-7–10, and there is similar equivalent series resistance (ESR) of about 0.4 Ω for RGO-7–10 and RGO at the high frequency region. However, the Nyquist plot of RGO-7–10 shows a much more vertical line at the low frequency region, indicating fast ion diffusion in the electrode material owing to its loose structure. Figure 7f exhibits the excellent cycling performance of the electrodes at different current densities of 10 A g^−1^, 20 A g^−1^, 30 A g^−1^ and 40 A g^−1^, with 5 000 cycles for each current density, respectively. Obviously, there is no degradation after 20 000 cycles for both samples.

## Discussion

**Figure 8:**
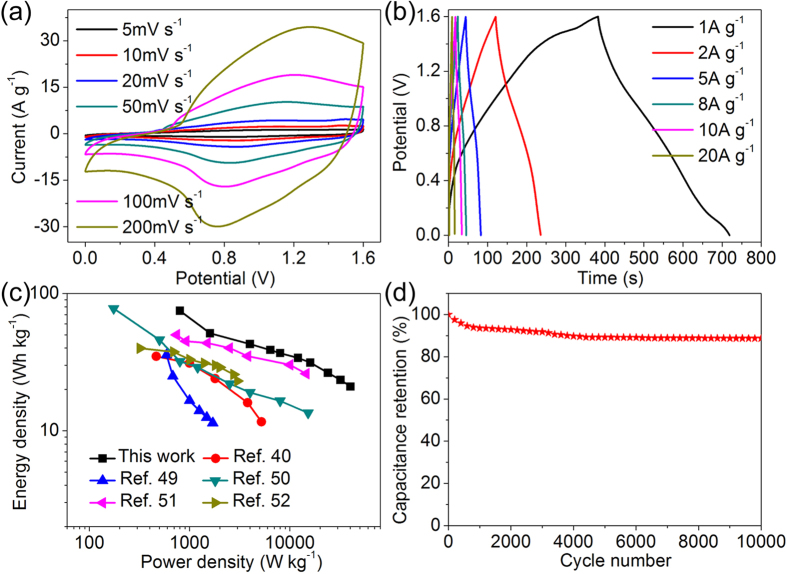
The electrochemical properties of the RGO-Ni(OH)_2_//RGO-7-10 ASC: (**a**) CV curves at different scan rates, (**b**) GCD curves at different current densities, (**c**) Ragone plots of different asymmetric supercapacitors, and (**d**) cycling performance measured at a current density of 15 A g^−1^. All data in this figure are based on the asymmetric device with total mass of both electrodes.

Based on above results, we can conclude that RGO-Ni(OH)_2_ (denoted as RGO-Ni(OH)_2_-2 in Supplementary Information) and RGO-7–10 exhibit the best electrochemical performance compared to other congener materials. To establish a practical energy store device, an ASC was assembled based on the RGO-Ni(OH)_2_ as the positive electrode active material and RGO-7–10 as the negative electrode active material. The asymmetric device is demonstrated in a voltage window of 1.6 V at different scan rates from 5 to 200 mV s^−1^ (Fig. 8a). The GCD curves of the as-fabricated ASC device at different current densities of 1 to 20 A g^−1^ are depicted in Fig. 8b. It is found that both the CV and GCD curves keep their characteristic profile without any polarization at a full voltage, revealing an excellent capacitive performance. Figure 8c shows the Ragone plots (energy density vs power density) of the as-fabricated ASC. It is clearly seen that the device displays an outstanding energy density of 75 Wh kg^−1^ at 800 W kg^−1^, approximately. More remarkably, even when the power density reaches 40 000 W kg^−1^, the energy density still maintains 21 Wh kg^−1^. The results are higher than the similar materials including NiCo_2_O_4_//AC (34.8 Wh kg^−1^ at a power density of 464 W kg^−1^)^36^, Ni(OH)_2_//AC (35.7 Wh kg^−1^ at a power density of 590 W kg^−1^)^45^, Ni(OH)_2_-G//PG (13.5 Wh kg^−1^ at a power density of 15 200 W kg^−1^)^46^ and NiO/AC//G (50 Wh kg^−1^ at a power density of 740 W kg^−1^)^47^, NiO//rGO (39.9 Wh kg^−1^ at a power density of 320 W kg^−1^)^48^. The superior performance is achieved mainly owing to the high capacitances of two electrodes, excellent rate capability and appropriate mass ratio of positive and negative electrode materials. Moreover, Fig. 8d shows the excellent cycling stability of the ASC with 89% capacitance retention after 10 000 cycles at a high current density of 15 A g^−1^. Therefore, the excellent electrochemical performance of as-built ACS indicates its attractive application in energy store system.

In summary, we have developed a novel strategy by taking advantage of synergistic effect between ultra-small Ni(OH)_2_ nanoparticles and RGO sheets to build a high-performance ASC. The incorporation of RGO sheets which serve as ideal substrate is able to anchor ultra-small Ni(OH)_2_ particles, resulting in effectively preventing the agglomeration of Ni(OH)_2_ particles. At the same time, ultra-small nanoparticles are able to act as separators between RGO layers to effectively reduce the irreversible layer-to-layer restacking. As a consequence, on one hand, the RGO-Ni(OH)_2_ composite exhibits high specific capacitance of 1717 F g^−1^ at 0.5 A g^−1^; on the other hand, the enhanced RGO exhibits excellent rate performance, even keeping 182 F g^−1^ at 100 A g^−1^. In addition, the oxide spacers are able to recycle for repeatedly preparing ultra-small Ni(OH)_2_ nanoparticles. More remarkably, the assembled ASC with RGO-Ni(OH)_2_ as the positive electrode and RGO-7–10 as the negative electrode possesses an outstanding energy density of 75 Wh kg^−1^ and an ultra-high power density of 40 000 W kg^−1^ and a robust cycle life. Therefore, the results presented here can provide useful information to take full advantage of Ni(OH)_2_ particles and RGO sheets for supercapacitor applications. In addition, the novel integrative route of designing electrode materials structure is an important reference to synthesize similar materials.

## Methods

### Synthesis of Reduced Graphene Oxide sheets

Graphene oxide (GO) was prepared from natural graphite powders (99.99%, 325 mesh, purchased from Qingdao Huatai Tech. Co., Ltd., China.) by a modified Hummers method^49^,^50^. Then reduced graphene oxide (RGO) was synthesized according a previous report with a little change^50^. In a typical synthesis process, 100 ml GO dispersion (about 1.0 mg ml^−1^) was heated to 95 ^o^C in a round bottom flask with magnetic stirring. After the suspension temperature reached 95 ^o^C, 1 ml hydrazine (80% wt%) and 100 μl ammonium hydroxide (28% wt%) were injected. The mixture was refluxed at 95 ^o^C for 1 hour with stirring. After by dialysis of the mixture dispersion with the cellulose ester membrane bag (MD77, 8000–14000) for one week to fully remove unreacted hydrazine and ammonia, the high quality RGO suspension (about 0.45 mg ml^−1^) can be obtained for further use. It is noted that the as-prepared RGO sheets are low degree of reduction, still having a number of residual oxygen-containing functional groups on their surfaces.

### Preparation of RGO-Ni(OH)_2_ Composite for Positive Electrode Material

The ultra-small Ni(OH)_2_ particles were synthesized based on our previously reports^51^,^52^. As shown in scheme 1a, the Ni(OH)_2_ and RGO-Ni(OH)_2_ composite were prepared via a simple two-step approach. Firstly, 1.0 g Ni(NO_3_)_2_·6H_2_O was dissolved in 300 ml de-ionized water with the aid of stirring at room temperature, then 3.0 g trisodium citrate hydrate was added. After the solution became transparent green, 0.8 g sodium borohydride was added to the mixture solution. For few minutes, the solution color became black, and the black solution kept in 40 °C bath for a few hours until the solution color turned to green. After this, the stable nickel-citrate specie was successfully formed to serve as Ni(OH)_2_ precursor for further use. Secondly, for the synthesis of RGO-Ni(OH)_2_ (with about 90 wt% Ni(OH)_2_, RGO-Ni(OH)_2_ denoted as RGO-Ni(OH)_2_-2, see Supplementary Table S1) composite with the optimal ratio between RGO and Ni(OH)_2_ for winning the best capacitance, 85.7 ml Ni(OH)_2_ precursor solution which can prepare 90.0 mg Ni(OH)_2_ approximately was dropwise added to 22.2 ml as-prepared RGO dispersion (about 0.45 mg ml^−1^), followed by stirring for 30 min and sonication for 15 min. Thus, the nickel-citrate specie was distributed on RGO surfaces. Subsequently, 0.27 g NaOH was added to the mixture under stirring to act as the precipitator, and the corresponding pH was about 13. The mixture dispersion was then transferred to a Teflon autoclave and maintained at 80 °C for 24 h. Finally, the resulting precipitate composite was washed several times with distilled water and proceeded freeze-drying. For comparison, pure Ni(OH)_2_ was synthesized under the same condition except for the addition of RGO. Also, the other RGO-Ni(OH)_2_ composite samples with different ratios between RGO and Ni(OH)_2_ were prepared using a similar method from the raw materials with different contents (see Supplementary table S1).

### Synthesis of Enhanced RGO for Negative Electrode Material

The enhanced RGO sheets were prepared as shown in scheme 1b. In a typical preparation, 200 mg GO suspension (1.0 mg ml^−1^) was further dispersed by sonication for 15 minutes. 133.3 ml Ni(OH)_2_ precursor solution was dropwise added to the GO dispersion via the aid of stirring. The pH of the solution was adjusted to 13 by adding NaOH. The mixture solution was transferred into a 500 ml Teflon-lined autoclave and heated for 24 h at 80 °C. After that, the GO-Ni(OH)_2_ composite can be obtained by washing with distilled water for several times, followed by freeze-drying. Then the composite powders were sealed in a tube and heated at 300 °C for 2 h under an argon atmosphere with a heating rate of 5 °C min^−1^. Finally, the enhanced RGO (denoted as RGO-7–10) sheets were obtained after hydrochloric acid treatment with the aid of stirring and sonication, followed by washing with distilled water and drying. It is worth noting that the recyclable nickel ions can be repeatedly used as nickel source in this experiment (see Supplementary Fig. S9). For comparison, the samples with different mass ratios between Ni(OH)_2_ and GO were prepared using the same method (see Supplementary table S2). The final samples were denoted as RGO-x-y (x and y represent the relative mass of Ni(OH)_2_ and GO, respectively). In addition, the conventional RGO sheets were also prepared by directly thermal treatment under the same condition (see Supplementary table S2).

### Morphology and Structural Characterization

Field emission scanning electron microscope (FESEM; JSM 6701F), transmission electron microscope (TEM; JEOL 2100 FEG), powder X-ray diffraction (XRD; Rigaku D/Max-2400, Cu-Kα radiation, λ = 0.15405 nm) and Raman spectroscope (JY-HR800, the excitation wavelength of 532 nm) were employed to investigate the morphology and structure of as-prepared electrode materials. X-ray photoelectron spectroscope (XPS, Physical Electronics, PerkinElmer PHI-5702) and Fourier transform infrared spectrometer (FTIR IFS120HR) were employed to examine the chemical species. Thermogravimetry (TG) measurements were performed by a thermo gravimetric analyser (TGA-STA 449C, from 30 °C to 800 °C in air). The nitrogen adsorption–desorption isotherm measurements were performed on a Micromeritics ASAP 2020 volumetric adsorption analyzer at 77 K. The Brunauer–Emmett–Teller (BET) method was utilized to calculate the specific surface area. The pore-size distribution was determined by a nonlocal density functional method using the adsorption data, and assuming a slit pore model.

### Electrode preparation and electrochemical measurements

The working electrodes were fabricated as follows: 85 wt% active material (Ni(OH)_2_, RGO-Ni(OH)_2_ or RGO) 10 wt% acetylene carbon, and 5 wt% polytetrafluoroethylene (PTFE) binder were firstly mixed well with ethanol to form a slurry. After briefly allowing the solvent to evaporate, the resulting paste was coated onto nickel foam, followed pressed at 10 MPa and dried for 10 h at 60 °C in air. It is noted that 5 mg Ni(OH)_2_ or RGO-Ni(OH)_2_ was used for preparing a working electrode, while 2 mg RGO-based was used for preparing a working electrode.

Electrochemical properties of each electrode material were investigated using an electrochemical working station (CHI660D, Shanghai, China) in a three-electrode system. A platinum gauze electrode (about 2 cm^2^) and a saturated calomel electrode (SCE) acted as the counter electrode and the reference electrode, respectively. An aqueous solution of KOH (2 M) was used as the electrolyte. The electrochemical impedance spectrum (EIS) measurements were carried out in the frequency range from 0.01 Hz to 100 kHz at open circuit potential with an ac perturbation of 10 mV. The average specific capacitance value (C_s_) was calculated from the galvanostatic discharge curve, using the following equation:


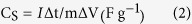


Where I is the constant discharge current (A), Δt represents the discharge time for a full discharge (s), m indicates the mass of the corresponding active material (g) and ΔV represents the potential range of a full discharge (V).

A two-electrode cell configuration was used to measure the performance of as- assembled asymmetric supercapacitor (ASC) in 2 M KOH electrolyte. To construct the ASC, the RGO-Ni(OH)_2_ (1.0 mg) and the RGO-7–10 (2.0 mg) were used as the positive electrode and the negative electrode, respectively. Their mass ratio is based on charge balance theory (q^+^ = q^−^)^52^. The energy density (E) of ASC was calculated by the specific capacitance (C) and the cell voltage (V) according to the following equation:


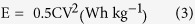


The power density (P) of ASC was achieved by the E and the discharging time (t) according to the following equation:


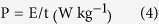


## Additional Information

**How to cite this article**: Liu, Y. *et al.* Synergistic Effect between Ultra-Small Nickel Hydroxide Nanoparticles and Reduced Graphene Oxide sheets for the Application in High-Performance Asymmetric Supercapacitor. *Sci. Rep.* **5**, 11095; doi: 10.1038/srep11095 (2015).

## Supplementary Material

Supplementary Information
