# Supplementary material for: Synergistic Effect between Ultra-Small Nickel Hydroxide Nanoparticles and Reduced Graphene Oxide sheets for the Application in High-Performance Asymmetric Supercapacitor
Source: Sci Rep. 2015 Jun 8;5:11095. doi: 10.1038/srep11095 (PMC4459188; doi:10.1038/srep11095)
Supplement: Supplementary Information [file srep11095-s1.pdf]

Supplementary Information for Scientific Reports

Synergistic Effect between Ultra-Small Nickel Hydroxide Nanoparticles and Reduced Graphene Oxide Sheets for the Application in High-Performance Asymmetric Supercapacitor

Yonghuan Liu[a,b], Rutao Wang[a] & Xingbin Yan[a*]

[a] *Laboratory of Clean Energy Chemistry and Materials, State Key Laboratory of Solid Lubrication, Lanzhou Institute of Chemical Physics, Chinese Academy of Sciences, Lanzhou 730000, P. R. China*

[b] *Graduate University of Chinese Academy of Sciences, Beijing 100080, P. R. China*

---

* Corresponding author. Tel.: +86 931 4968055; fax: +86 931 4968055.

E-mail address: xbyan@licp.cas.cn (X. B. Yan).

Positive electrode materials of $Ni(OH)_2$ and RGO-$Ni(OH)_2$ composite:

Table S1. The volume contents of raw materials and the carbon contents in final RGO-$Ni(OH)_2$ composites.

Samples	$Ni(OH)_2$ precursor volume (ml)	RGO dispersion volume (ml)	final carbon content mass (%)
$Ni(OH)_2$	95.2	0	2.7
RGO-$Ni(OH)_2$-1	90.5	11.1	6.5
RGO-$Ni(OH)_2$-2	85.7	22.2	14.0
RGO-$Ni(OH)_2$-3	80.9	33.3	18.7
RGO-$Ni(OH)_2$-4	76.2	44.4	26.3
RGO-$Ni(OH)_2$-5	66.7	66.7	32.4

In our system, per-volume unit $Ni(OH)_2$ precursor solution contains approximate 1.05 mg $Ni(OH)_2$ and the concentration of RGO dispersion is about 0.45 mg ml^{-1}. The carbon content was measured via elemental analyzer.

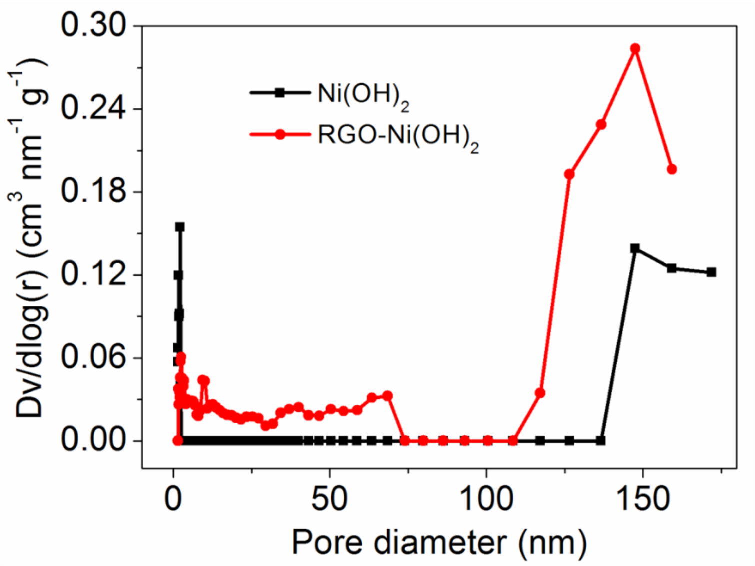

Figure S1. Pore size distribution curves of pure $Ni(OH)_2$ and RGO-$Ni(OH)_2$ (RGO-$Ni(OH)_2$-2) composite.

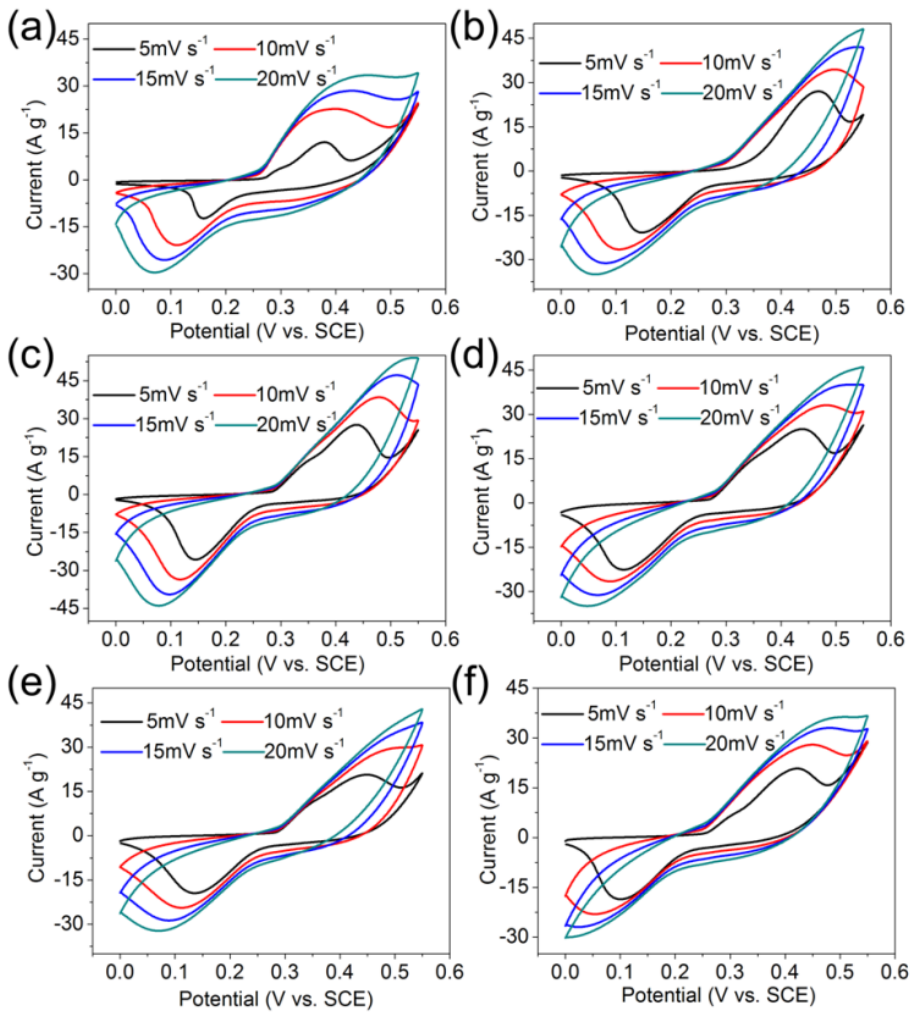

Figure S2. (a) CV curves of a series of RGO-Ni(OH)$_2$ composites at different scan rates: (a) pure Ni(OH)$_2$, (b) RGO-Ni(OH)$_2$-1, (c) RGO-Ni(OH)$_2$-2, (d) RGO-Ni(OH)$_2$-3, (e) RGO-Ni(OH)$_2$-4 and (f) RGO-Ni(OH)$_2$-5.

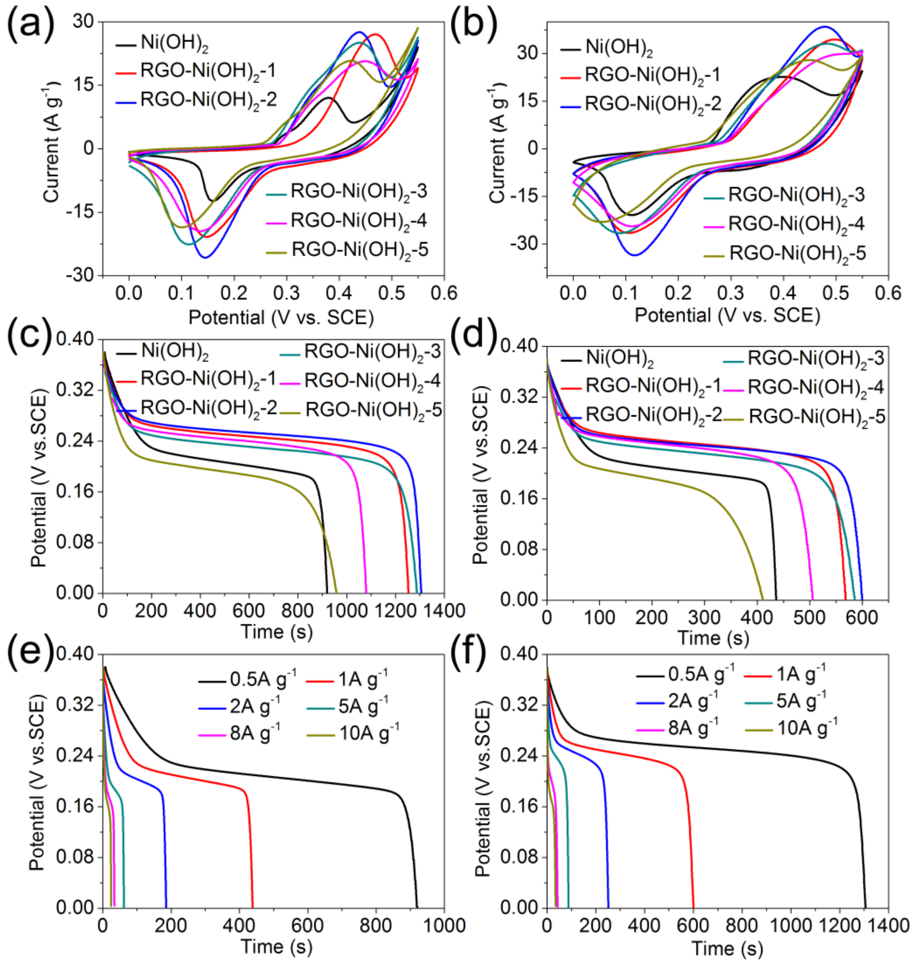

Figure S3. CV curves of a series of RGO-Ni$(OH)_2$ composites: (a) at a scan rate of 5 mV s^{-1} and (b) at a scan rate of 10 mV s^{-1}; galvanostatic discharge curves of a series of RGO-Ni$(OH)_2$ composites: (c) at a current density of 0.5 A g^{-1} and (d) at a current density of 1 A g^{-1}; (e) galvanostatic discharge curves of pure Ni$(OH)_2$ at different current densities. (f) galvanostatic discharge curves of RGO-Ni$(OH)_2$ (RGO-Ni$(OH)_2$-2) composite at different current densities.

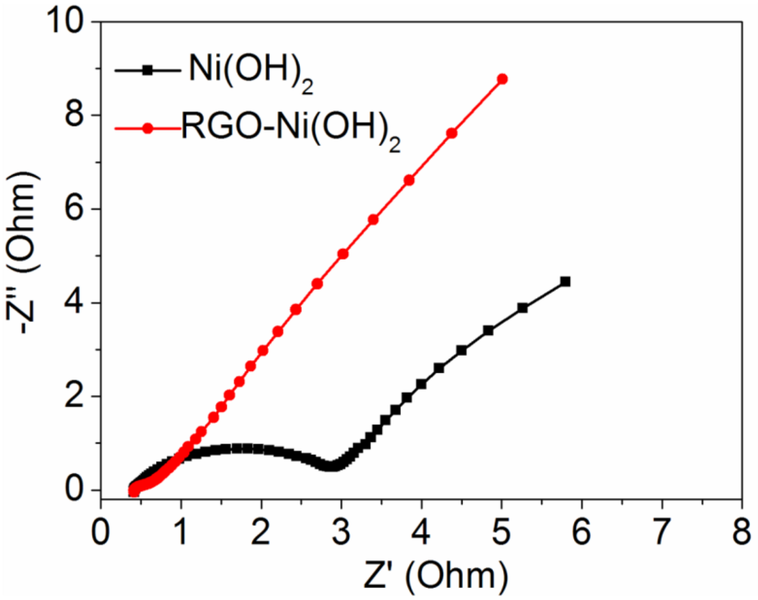

Figure S4. Nyquist plots of pure $Ni(OH)_2$ and RGO-$Ni(OH)_2$ (RGO-$Ni(OH)_2$-2) composite.

Negative electrode materials of RGO and enhanced RGO

Table S2. The volume contents of raw materials, and I_D/I_G values and O, N contents in final RGO samples.

Samples	$Ni(OH)_2$ precursor volume (ml)	GO dispersion volume (ml)	I_D/I_G	O (at%)	N (at%)
RGO	0	200	1.15	16.62	1.74
RGO-2-10	38.1	200	1.25	18.50	2.06
RGO-5-10	95.2	200	1.39	18.88	2.16
RGO-7-10	133.3	200	1.50	18.20	2.11
RGO-10-10	190.5	200	1.54	19.34	1.68
RGO-15-10	285.7	200	1.60	17.20	2.22

In this system, per-volume unit $Ni(OH)_2$ precursor solution contains approximate 1.05 mg $Ni(OH)_2$, and the concentration of GO dispersion is about 1.0 mg ml^{-1}. The oxygen and nitrogen contents were quantified by XPS. The relative high oxygen content is owing to low thermal treatment temperature.

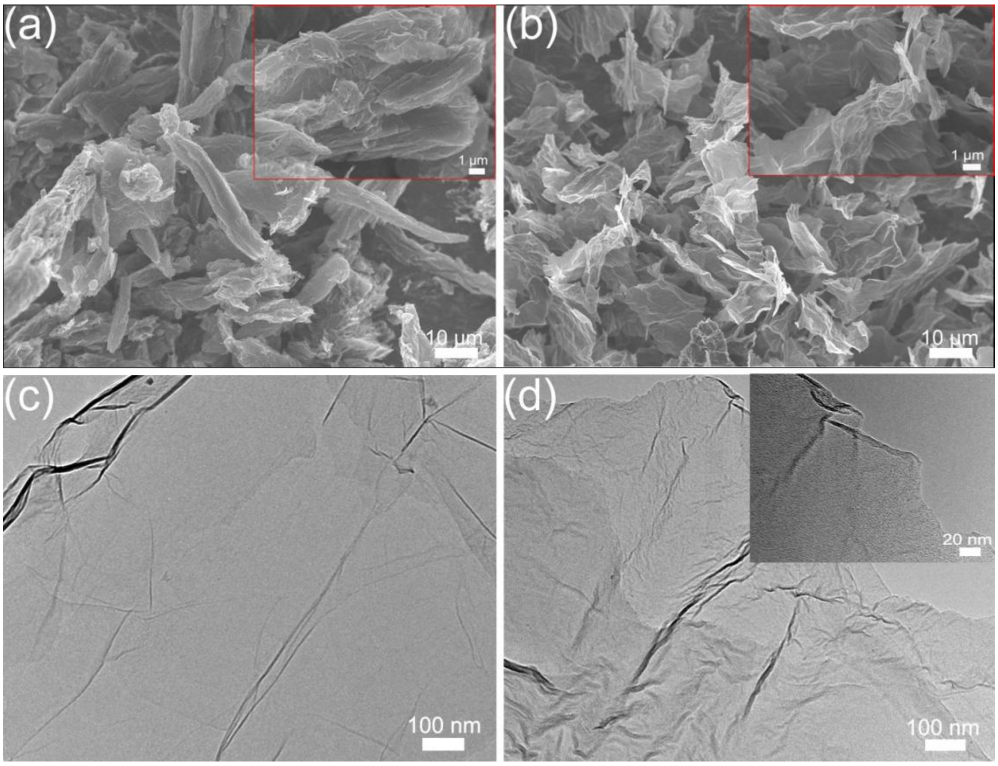

Figure S5. Low and high (inset) magnification SEM images of (a) RGO prepared by direct thermal reduction and (b) enhanced RGO-7-10 prepared with aid of $Ni(OH)_2$. (c) and (d) represent the corresponding TEM images, respectively.

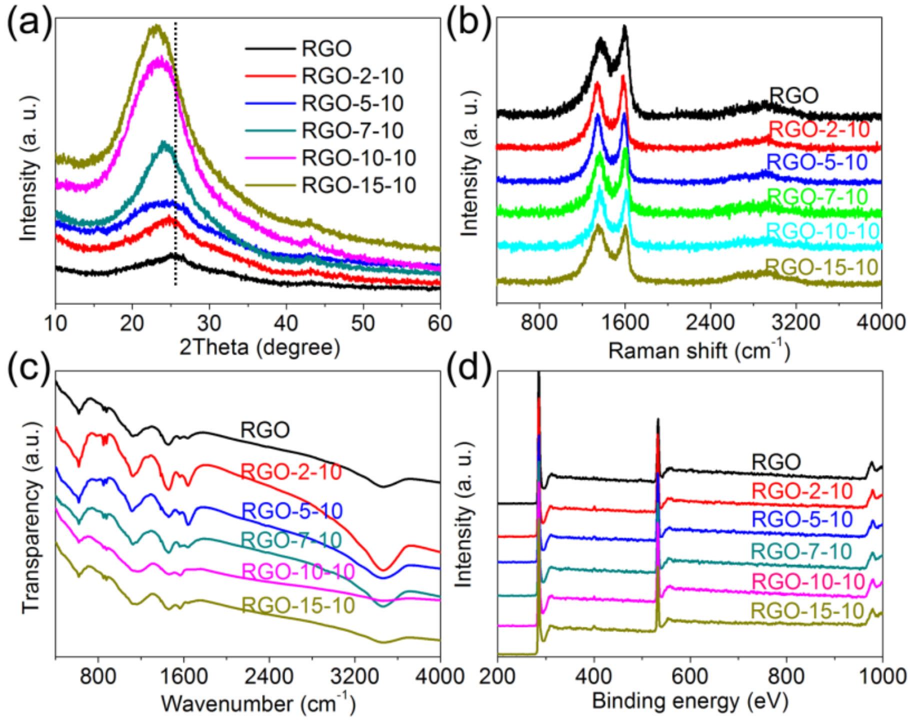

Figure S6. The XRD patterns (a), Raman spectra (b), FTIR spectra (c) and XPS spectra (d) of a series of RGO samples.

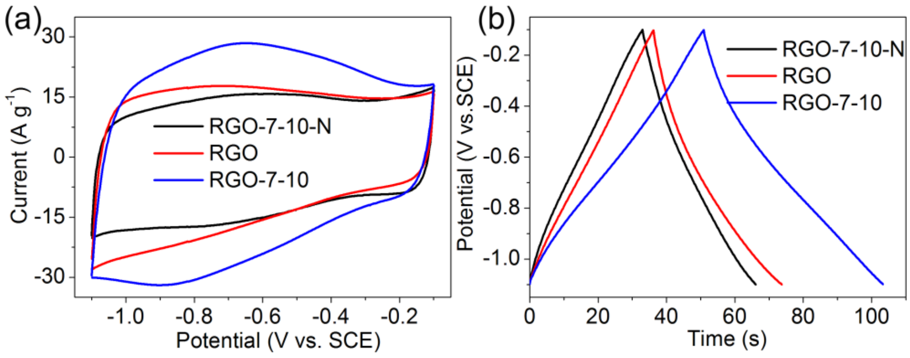

Figure S7. (a) CV curves of pure RGO prepared by direct thermal reduction and enhanced RGO prepared with aid of $Ni(OH)_2$ at a scan rate of 100 mV s^{-1}. Here RGO-7-10 is the sample after HCl etching and RGO-7-10-N is the sample before HCl etching (containing NiO). (b) GCD curves of RGO, RGO-7-10 and RGO-7-10-N samples at a current density of 5 A g^{-1}. The specific capacitance of RGO-7-10 is higher than RGO-7-10-N mainly due to hardly capacitance contribution of NiO at this potential window of -1.1 V to -0.1 V.

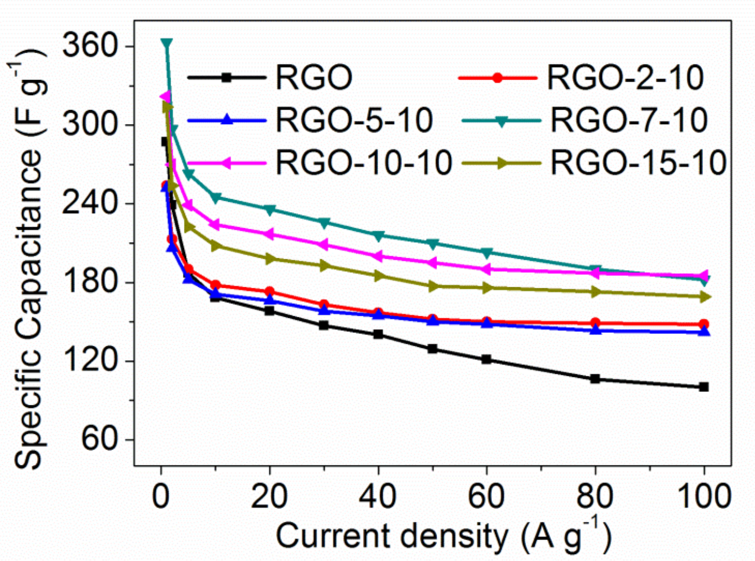

Figure S8. The specific capacitance of a series of RGO samples as a function of current density.

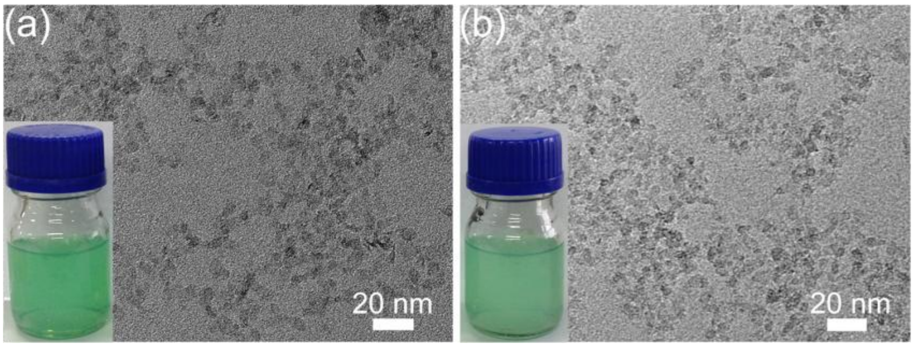

Figure S9. (a) TEM image of $Ni(OH)_2$ nanoparticles obtained from fresh chemical reagents.(b) TEM image of $Ni(OH)_2$ nanoparticles obtained from reclaimed nickel ions. Insets are the corresponding photographs of $Ni(OH)_2$ aqueous dispersions.

Recycling nickel ions: When the NiO nanoparticles attached on RGO sheets were removed by HCl etching, the reaction solution containing Ni^{2+} could be separated from RGO by sonication and filtration. The pH value of the reclaimed solution was adjusted to ~6 by adding NaOH. After that, nickel dichloride could be obtained by recrystallization, which can be repeatedly used as nickel source to prepare ultra-small $Ni(OH)_2$ nanoparticles. The following processes are similar to above mention in **methods section**.
